# Efficient and Safe Knockout of *AR* and *DMRT1* Mediated by Cytosine Base Editors in Chicken DF-1 and PGCs

**DOI:** 10.3390/vetsci13050455

**Published:** 2026-05-06

**Authors:** Xiaori Gao, Na Tang, Zhifeng Zhao, Yanhua He, Yitong Shen, Xian Zou, Chenglong Luo

**Affiliations:** 1 College of Animal Science and Technology, Foshan University, Foshan 528225, China; 18437339980@163.com; 2 Key Laboratory of Agricultural Animal Genetics, Breeding and Reproduction, Ministry of Education, Huazhong Agricultural University, Wuhan 430070, China; tna@webmail.hzau.edu.cn (N.T.); shenyitong@webmail.hzau.edu.cn (Y.S.); 3 Guangdong Key Laboratory of Animal Breeding and Nutrition, Guangdong Academy of Agricultural Sciences, Institute of Animal Science, Guangzhou 510640, China; zhifengzhao95@163.com (Z.Z.); heyanhua1111@163.com (Y.H.)

**Keywords:** cytosine base editor (CBE), *AR* gene, *DMRT1* gene, chicken, primordial germ cells (PGCs), gene knockout, sgRNA screening, avian biotechnology

## Abstract

This study aimed to establish an efficient and precise cytosine base editor (CBE)-mediated knockout system in chicken somatic cells and primordial germ cells (PGCs). PGCs are pivotal for generating genome-edited chickens, but low transfection efficiency limit their application. Unlike CRISPR/Cas9, CBEs achieve precise C-to-T conversion without DNA double-strand breaks or donor templates, making them safer for avian genome engineering. We used CBEs to introduce premature stop codons in exon 1 of the sex-determining *AR* and *DMRT1* genes for targeted knockout. Among 12 screened sgRNAs, sgRNA6 (*AR*, 94.67 ± 6.66%) and sgRNA9 (*DMRT1*, 6.67 ± 6.51%) performed best in DF-1 cells; in PGCs, their editing efficiencies reached 51.0% and 91.0%, respectively. No off-target mutations were detected in edited DF-1 cells. These findings confirm that CBE-mediated knockout is highly efficient and safe in chicken somatic and germ cells, providing a robust tool for avian genome editing.

## Simple Summary

This study aimed to develop a highly efficient and accurate single-base gene editing and knockout system in chicken body cells and reproductive stem cells. Reproductive stem cells are essential for creating gene-edited chickens, but their low transfection efficiency has limited real-world application. Unlike traditional gene editing tools that may damage DNA structure, the cytosine base editing technique used here modifies single DNA letters precisely without breaking DNA strands, making it much safer for chicken genetic modification. We targeted two key genes that control chicken sex development, added early stop signals to turn off these genes, and screened out the most effective editing guides. This system showed high editing efficiency in both chicken body cells and reproductive stem cells, and no unwanted off-target changes were detected in body cells. Our work provides a safe and reliable gene editing tool for poultry genetic research and precision breeding, supporting the advancement of avian biotechnology and sustainable livestock farming.

## 1. Introduction

The development of genome-edited chickens has undergone technological advancements from random transgene integration to precise targeted editing. Currently, CRISPR/Cas9 is the mainstream technology for generating gene-edited chickens, often integrated with PGC culture and transplantation systems or sperm transfection to enable efficient genome editing [1,2,3]. PGCs are pluripotent germline progenitor cells that circulate in the embryonic bloodstream at embryonic day 2.5, migrate to the developing gonads, and ultimately differentiate into mature ova or spermatozoa in adult chickens [4,5,6]. To generate genome-edited chickens, PGCs can be isolated, cultured, genetically modified ex vivo, and re-implanted into recipient embryos [7], or directly edited in vivo during their circulation in the embryonic bloodstream [8]. Modified PGCs can then stably transmit the edited genetic traits to offspring through gametogenesis [9]. However, efficient DNA delivery into PGCs remains challenging due to low transfection efficiency, limiting the production of genetically modified chickens [10,11]. At present, stable culture systems for chicken PGCs have been widely established in laboratories [12]. Furthermore, our laboratory has previously resolved the bottleneck of low transfection efficiency in PGCs, laying a solid foundation for the rapid generation of gene-edited chickens [10]. To date, CRISPR/Cas9 remains the dominant gene-editing tool used in chickens, while other advanced editing systems have rarely been reported. Compared with the CRISPR/Cas9 system, base editors (BEs) represent a more precise and safer gene-editing tool with great potential in poultry genetic improvement.

Base editors (BEs) are innovative gene-editing tools developed by fusing the CRISPR/Cas system with a single-strand deaminase. They enable precise single-base substitutions at the DNA or RNA level, and can efficiently converted one base pair to another in both dividing and non-dividing cells. Mose importantly, base editors function without inducing double-strand breaks (DSBs) in genomic DNA or requiring exogenous donor DNA templates, thus greatly improving editing safety. Base editors are mainly classified into two categories: CBEs and adenine base editors (ABEs) [13]. CBEs consist of nCas9, cytosine deaminase, and uracil glycosylase inhibitors (UGIs), facilitating efficient cytosine to thymine (C-to-T) conversions [14,15]. In addition to these well-established platforms, a diverse array of emerging base editing variants has been rapidly developed to address broader sequence constraints and achieve distinct editing outcomes, such as glycosylase base editors (GBEs) that repurpose uracil DNA glycosylase (UNG) to facilitate C → G transversions, dual base editors (DBEs) that enable concurrent C → T and A → G edits [16,17]. Base editing (BE) technologies have been successfully extended from initial validation in human cell lines and model organisms such as zebrafish and mice to important agricultural species including pigs, sheep and cattle, improving growth and product quality. In contrast, the application of base-editing systems in avian species, especially chickens, remain relatively limited [18,19,20,21,22].

Given that base editing offers more precise genome editing and greater safety for livestock genetic improvement, this study aimed to employ CBE to generate *AR* and *DMRT1* knockout chicken cells. We focused on two key genes in avian sex determination and gonadal development: the androgen receptor (*AR*), which regulates androgen signaling and is critical for male development and doublesex and mab-3 related transcription factor 1 (*DMRT1*), which regulates gonadal differentiation, gametogenesis, and sex determination across multiple species. To date, no studies have systematically achieved precise knockout of chicken *AR* and *DMRT1* using CBEs.

In response to the limited application of base editing in avian species, the core purpose of this study was to establish an efficient and precise CBE-mediated knockout system for chicken somatic cells and PGCs, and to use this system to achieve the first systematic CBE-based knockout of the chicken *AR* and *DMRT1* genes. We demonstrated that CBEs can efficiently introduce premature stop codons in these genes in both DF-1 cells and PGCs, generating edited cell lines that confirm the efficacy of this approach. This work provides a reliable technical basis for subsequent gene editing in chicken PGCs and the construction of in vivo gene-edited models, highlighting the great application potential of base-editing technology in avian biotechnology and genetic breeding.

## 2. Materials and Methods

### 2.1. sgRNA Design and Screening for CBE-Mediated Gene Knockout

All sgRNAs were designed according to the requirements of the CBE system. Single-guide RNA (sgRNA) design requires retrieving the reference sequence of the *AR* gene from the authoritative database NCBI first. The exon 1 of the *AR* (NCBI gene ID: NC_052535.1) gene and the *DMRT1* (NCBI gene ID: NC_052572) genes were obtained from the NCBI website and the sequences of introns and exons of *AR* and *DMRT1* were determined. Designing sgRNAs for each gene using the online tool CRISPOR v5.2 (http://crispor.tefor.net/, accessed on 4 October 2025). Input the target sequence, select the species genome and the corresponding PAM (20 bp-NGN-SpG), and all potential sgRNAs on the target sequence can be obtained. These sgRNAs are then screened, and sgRNAs containing CAG, CAA, or CGA in the sequence are preferentially selected. Finally, from these sgRNAs, appropriate sgRNAs are selected according to specificity, cleavage efficiency scores, Off-Target scores, etc. These selected sgRNAs were validated as the final designed sgRNAs targeting exon 1 of the *AR* gene, and were applied for subsequent gene editing experiments using the QH173 all-in-one vector, which co-expresses both SpGCas9 protein and the target sgRNA. Corresponding sgRNAs were designed against exon 1 of the chicken *AR* and *DMRT1* genes. The corresponding information of all sgRNAs are shown in Table 1.

### 2.2. Plasmid Construction

**Figure 1:**
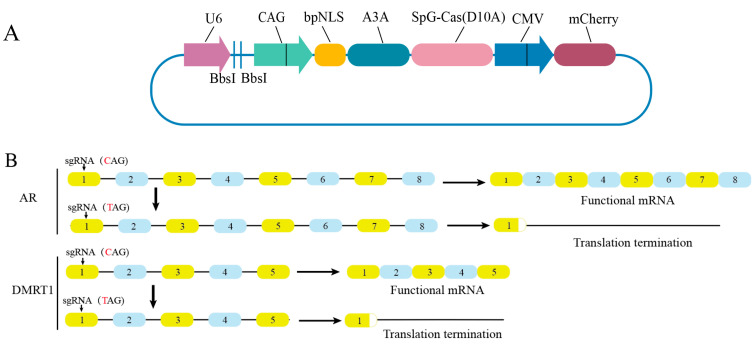
Schematic of CBE vectors and target sites for *AR* and *DMRT1* editing. (**A**) Schematic of the CBE expression vector. sgRNA and CBE are driven by the U6 and CMV promoters, respectively; mCherry is expressed for transfected cell screening. (**B**) Target editing sites in *AR* and *DMRT1*. CBE-mediated C → T conversion in exon 1 generates a premature stop codon to induce gene knockout.

The design and construction of the CBE plasmid were performed. One-step method was used to ligate sgRNA into the editing plasmid QH173 (Figure 1A). (1) After the synthesis of the two oligonucleotides, they were annealed into double strands using T4 Buffer [23], (2) using T4 DNA ligase and restriction endonuclease BbsI (Thermo, Waltham, MA, USA), each sgRNA was inserted individually into the BbsI sites of the backbone vector QH173, and (3) following ligation, the products which was then transformed into DH5α (WEIDI Shanghai, China), plated on LB containing ampicillin, and cultured and sequenced using single colonies. After the correct sequence was obtained, the culture was expanded. Finally, ZXP1-12 plasmids were extracted using an endotoxin-free medium plasmid extraction kit (Omega, Beijing, China), and the purified plasmids were used for cell [24,25]. Previous experiments in this laboratory have found that the CMV promoter exhibits poor expression efficiency in PGCs. Using plasmids sgRNA6 and sgRNA9 as templates, the vector backbones were recovered by double digestion with the restriction endonucleases SalI and NotI (Thermo, Waltham, MA, USA), respectively. Meanwhile, the PGK-EGFP fragment was amplified by PCR with primers designed from the PGK-EGFP-Donor plasmid and subsequently purified. The purified backbones and PGK-EGFP fragments were ligated at a molar ratio of 1:3 to 1:10, with T4 DNA ligase added at a volume accounting for half of the total ligation mixture. The resulting recombinant plasmids were designated ZXP6-EGFP and ZXP9-EGFP. Primers are listed in the Supplemental Table S1.

### 2.3. Cell Culture

The chicken DF-1 cell line was maintained in Dulbecco’s Modified Eagle Medium (DMEM, Gibco, Grand Island, NY, USA) supplemented with 10% (*v*/*v*) fetal bovine serum (FBS, Sciencell, Carlsbad, CA, USA), 100 IU/mL penicillin, and 100 μg/mL streptomycin, and cultured in a cell incubator at 37 °C with 5% CO_2_. The cells were passaged every 2–3 days. DF-1 cells were seeded into 6-well plates and used for transfection 24 h later.

The PGCs medium composition was adapted from a previous study with slight modifications. The culture medium supplemented with 1× B-27 (17504044, Thermo Fisher Scientific, Waltham, MA, USA), 1× nonessential amino acids (11140050, Thermo Fisher Scientific, Waltham, MA, USA), 0.1 mM β-mercaptoethanol (ES-007-E, Sigma Aldrich, St Louis, MO, USA), 1× nucleosides (12571063, Thermo Fisher Scientific, Waltham, MA, USA), 0.15 mM CaCl_2_ (C7902, Sigma Aldrich, St Louis, MO, USA), 0.01% sodium heparin (H3149, Sigma Aldrich, St Louis, MO, USA), 0.2% ovalbumin (S7951, Sigma Aldrich, St Louis, MO, USA), 5 µg/mL OT (C7786, Sigma Aldrich, St Louis, MO, USA), 4 ng/mL FGF2, 25 ng/mL Activin A (120-14, PeproTech, Rocky Hill, NJ, USA) and 1% KOSR (10828028, Thermo Fisher Scientific, Waltham, MA, USA). PGC cultures were maintained at 37 °C in an atmosphere containing 5% CO_2_. PGCs were seeded into 6-well plates and used for transfection [3].

### 2.4. Cell Transfection

Approximately 24 h prior to transfection, DF-1 cells were passaged and seeded into 6-well plates at a confluency of 60–70%. Liposome-mediated transfection was performed using the Lip3000 Transfection Kit (Vazyme, Nanjing, China). At 6–12 h post-transfection, the medium was replaced with fresh complete medium (DMEM supplemented with 10% FBS) to provide sufficient nutritional support for the cells. Fluorescence was observed at 24 h post-transfection to preliminarily evaluate the transfection efficiency.

The transfection of PGCs was referenced to an article published by our laboratory with minor modifications [10]. PGCs were cultured in a feeder-free complete medium and seeded at 30–50% density in T75 culture flasks. The cells were incubated at 37 °C in a 5% CO_2_ incubator for 24 h to reach a 70–90% density before being harvested for transfection experiments. Transfection of Lonza electroporation system. The cells were transferred into a centrifuge tube and centrifuged at 200× *g* for 3 min. The supernatant was removed, and the cell pellet was resuspended in 2 mL PBS for counting. Cells were counted as described above. The PBS was removed after centrifuging again at 200× *g* for 3 min. A total of 3 × 10^6^ PGCs were resuspended in 100 µL of Entranster™-E electroporation buffer (98668-20, Engreen, Beijing, China) mixed with varying amounts of plasmid. The transfection mixture was then transferred into an electroporation cuvette and subjected to electroporation using the Lonza AAD-1001S Nucleofector^®^ system (Lonza, Baden-Wurttemberg, Germany). Post electroporation, PGCs were seeded into 6-well plates and maintained in complete medium at 37 °C.

### 2.5. Flow Cytometry

Following a 48 h transfection period, DF-1 cells were digested with 0.25% trypsin-EDTA (Thermo Fisher Scientific, Waltham, MA, USA), and the digestion was terminated by adding stop medium containing 10% FBS (Servicebio, Wuhan, China). Collect the cell suspension. Subsequently, the cell suspension was centrifuged at 600× *g* for 5 min; the supernatant was discarded, the collected cell pellet was resuspended in sterile PBS bufferto remove residual medium components. Finally, the top 5–10% of cells with the highest positive rate were sorted using a multi-functional automatic flow cytometry analyzer (FACS, Sony SH800, Sony Corporation, Tokyo, Japan). This instrument is characterized by high sorting purity (>98%) and excellent fluorescence resolution, and the sorted cells can be directly used for subsequent experiments. PGCs were harvested 200× *g* for 3 min and washed twice with PBS. Cells were transferred to BD tubes (Becton Dickinson, Franklin Lakes, NJ, USA) for detection of the fluorescent signal by flow cytometry using the Sony FACS. Data were then analyzed with the FlowJo V10 software [26].

### 2.6. Genotyping and Sequencing

PCRs were performed with KOD One™ PCR Master Mix—Blue—(TOYOBO) according to the manufacturer’s protocol, with the following primers (5′→3′) and PCR settings: *AR*: GAGCACCTCCCGGCCCAACT (forward), TCTCGACCGCCAGCCCCAT (reverse), annealing at 68 °C for 34 cycles; *DMRT1*: CGGGAGCGTAGGGACGGA (forward), AAAGCAGAAGCACGGTCAGATGTT (reverse), annealing at 64 °C for 34 cycles. Each PCR contained a water control. PCR samples were loaded on a 1.5% TBE-gel with a 2 kb DNA Marker (Trans). The observed bands were as expected, 571 bp for *AR*, 805 bp for *DMRT1*. Gel electrophoresis was employed to analyze the PCR products, followed by sequencing the insertion site at Tianyihuiyuan Company (Beijing, China) for further analysis.

### 2.7. Off-Target Site Prediction and Validation

For the sgRNA6 (targeting the *AR* gene) and sgRNA9 (targeting the *DMRT1* gene), potential off-target sites were predicted using the Cas-OFFinder online tool (RGENOME.net web server, https://www.rgenome.net/cas-offinder/, accessed on 15 September 2025) with the following parameters: number of mismatches = 3, DNA bulge size = 1, and RNA bulge size = 1. Based on the homology to the target sequence and genomic conservation, the top 18 high-risk off-target sites (including the sgRNA-binding regions and flanking sequences) were selected for validation.

Genomic DNA (gDNA) was extracted from flow cytometry-sorted CBE-edited DF-1 cells (experimental group) and wild-type (WT) DF-1 cells (negative control) using the One Step Mouse Genotyping Kit (Vazyme Biotech Co., Ltd., Nanjing, China). Specific primers were designed for each candidate off-target site (Supplementary Table S2) to amplify 500–600 bp genomic fragments, covering the sgRNA-binding region, protospacer adjacent motif (PAM), and upstream/downstream flanking sequences.

PCR amplification was performed using high-fidelity KOD FX enzyme (TOYOBO) in a 30 μL reaction system containing 100 ng of gDNA, 0.9 μL of each forward and reverse primer (10 μmol/L), 15 μL of 2× PCR buffer, 6 μL of dNTPs (2 μmol/L), and 0.6 μL of KOD FX enzyme. The amplification program was set as follows: initial denaturation at 94 °C for 2 min; 35 cycles of denaturation at 98 °C for 10 s, annealing at 60 °C for 30 s, and extension at 68 °C for 1 min; and a final extension at 68 °C for 5 min.

After verifying the specificity of PCR products via agarose gel electrophoresis, sanger sequencing was conducted. Sequencing results were aligned with the chicken reference genome (Galgal6) using SnapGene v5.2 (https://www.SnapGene.com, accessed on 11 October 2025) software. Peak profiles of the experimental group and control group were compared to analyze insertions/deletions (indels), C-T transitions, and other base substitutions at the off-target sites and their adjacent regions, thereby evaluating the editing specificity of CBE.

### 2.8. Analysis of Editing Efficiency

Previous studies have shown that the CBE editing window is located 4–8 nucleotides upstream of the PAM sequence, we focused our analysis on C-to-T substitutions within this window that generate the desired stop codon (CAG → TAG). sanger sequencing data were processed as follows: for each sequenced sample, the Applied Biosystems Sequence Trace (ab1) files along with the 20 bp sgRNA sequences were imported into the online tool EditR v1.0.10 (https://moriaritylab.shinyapps.io/editr_v10/, accessed on 15 December 2025) to determine the editing efficiency at the target loci. All experiments in DF-1 cells were performed with three biological replicates. Due to the technical challenges of primary chicken PGCs culture and transfection, the editing efficiency assay in PGCs was performed with two independent biological replicates. The editing efficiency values for all replicates were exported and organized into tables using Microsoft Excel. All editing efficiency results were statistically analyzed and graphically presented using GraphPad Prism software (GraphPad Prism 9, accessed on 1 February 2026). All data are presented as the mean ± standard deviation (SD). One-way analysis of variance (ANOVA) was used for comparisons among multiple groups. Statistical significance was defined as *p* ≤ 0.05 *, *p* ≤ 0.01 **, or *p* ≤ 0.001 ***, n.s.: not significant.

## 3. Results

### 3.1. High Editing Efficiency sgRNA Screening at Cell Level Mediated by CBE

To generate *AR* and *DMRT1* knockout cells, we employed the CBE system to target the *AR* and *DMRT1* genes in chicken. A single all-in-one vector was constructed through the integration of gRNA and CBE expression modules into the QH173 plasmid backbone (Figure 1A), and all plasmids were verified successfully by sanger sequencing. Twelve specific sgRNAs, named sgRNA1–8 and sgRNA9–12 (Table 1), were designed targeting exon 1 of the *AR* and *DMRT1* gene, respectively (Figure 1B). For *AR* and *DMRT1* knockout, CBE acts by altering the glutamine codon (CAG) to a premature stop codon, thereby inducing premature translation termination and gene silencing.

### 3.2. Verification of Plasmid Transfection and Positive Cell Screening in DF-1 Cells

**Figure 2:**
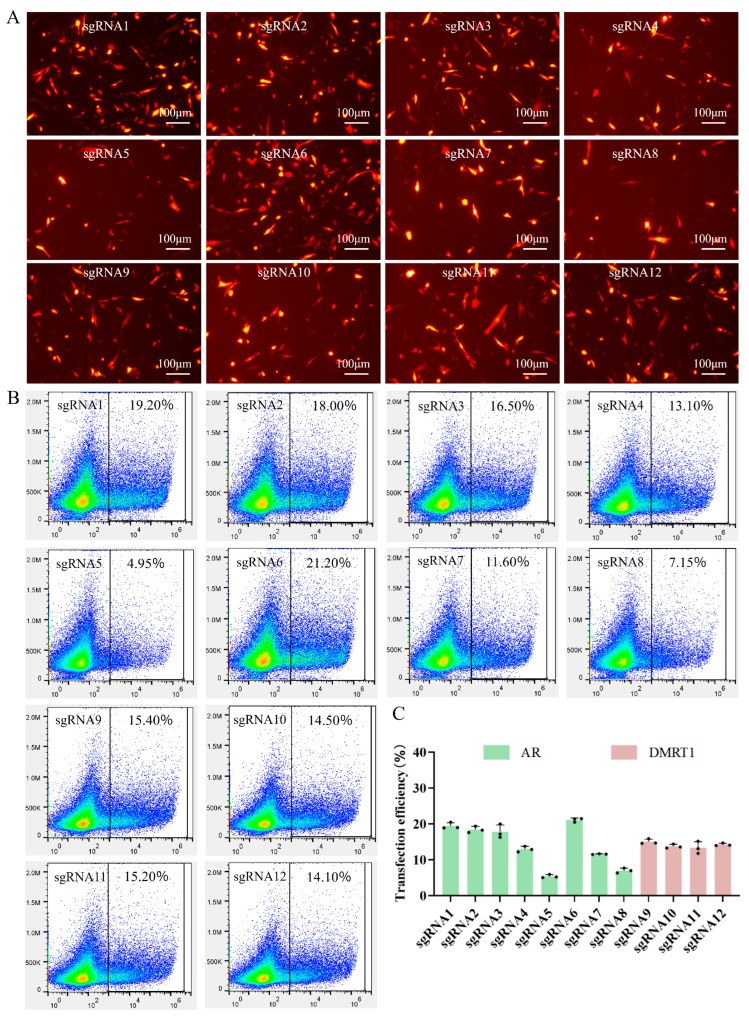
Experimental Design and Validation of Gene Editing Mediated by the CBE. (**A**) Fluorescence microscopy images showing the expression of mCherry red fluorescent protein in DF-1 cells transfected with 12 different sgRNA plasmids targeting *AR* (sgRNA1–8) and *DMRT1* (sgRNA9–12). Scale bars are omitted as representative fields are shown. (**B**) Representative flow cytometry dot plots quantifying the percentage of mCherry-positive cells for each sgRNA construct. The numbers indicate the positive rate of the gated cell population. (**C**) Quantitative analysis of transfection efficiency for all sgRNA plasmids. The transfection efficiency is presented as the mean ± standard deviation (SD) of three independent experiments. Green bars represent sgRNAs targeting *AR*, and maroon bars represent sgRNAs targeting *DMRT1*.

The constructed plasmids harboring the mCherry gene were transfected into DF-1 cells. Fluorescence microscopy was employed to observe the transfected cells (Figure 2A). The same group of cells was examined under two conditions: without fluorescence excitation (bright-field [BF] group) and with fluorescence excitation (mCherry group). Under fluorescence excitation, distinct red fluorescent signals were clearly visible, offering strong evidence for the successful transfection of DF-1 cells and the efficient, stable expression of the mCherry fluorescent protein. The intensity and distribution of the fluorescence further supported the high transfection efficiency.

FACS was then used to evaluate the transfection efficiency of transfected cells (Figure 2B). In the mCherry group, a clear population of cells with strong mCherry fluorescence was identified. Cells with the top 5–10% mCherry positivity rates were sorted for subsequent experiments. These results demonstrate that the liposome- mediated transfection method followed by flow cytometric sorting can effectively isolate mCherry—positive, successfully transfected cells from the total cell population, providing a sufficient number of target cells for subsequent studies.

To quantitatively compare the transfection performance across all designed sgRNAs, the transfection efficiencies of the 12 sgRNA constructs were further analyzed and summarized in a bar chart (Figure 2C). As illustrated, the transfection efficiencies varied among different sgRNAs, with sgRNA6 yielding the highest efficiency (21.20%), followed by sgRNA1 (19.20%) and sgRNA2 (18.00%). In contrast, sgRNA5 exhibited the lowest transfection rate (4.95%), suggesting notable sequence-dependent differences in delivery and expression efficiency. For the two target genes, *AR*-directed sgRNAs (sgRNA1–8) displayed overall robust transfection capabilities, while the four *DMRT1*-targeting sgRNAs (sgRNA9–12) also achieved favorable and stable efficiencies, ranging from 14.10% to 15.40%. Collectively, these data validate that most sgRNAs can be efficiently delivered and expressed in DF-1 cells, and the top-performing sgRNAs can be prioritized for subsequent gene-editing and functional validation assays.

### 3.3. Efficient sgRNA Screening

**Figure 3:**
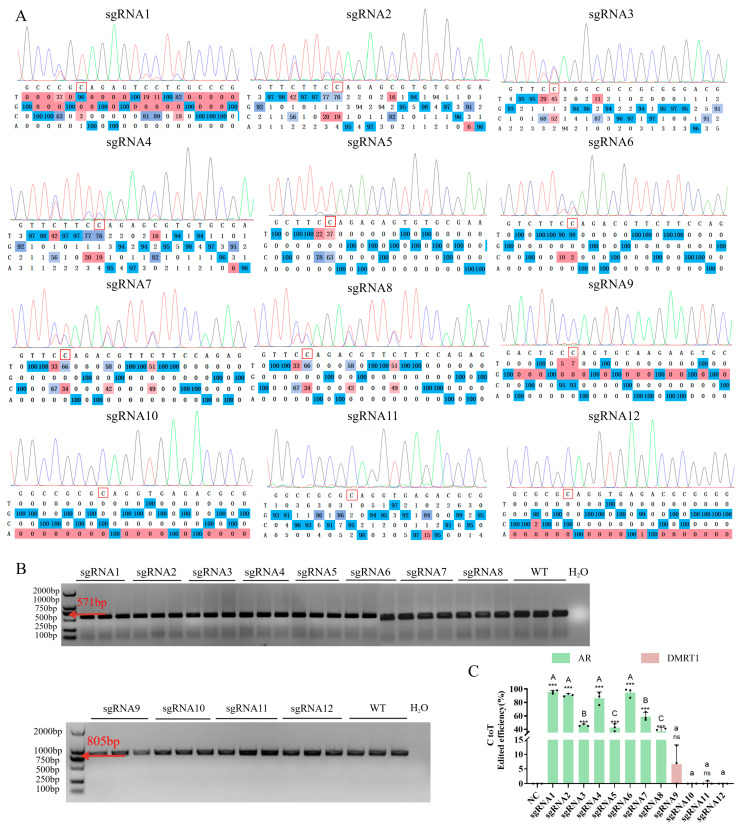
CBE editing efficiency at target loci. (**A**) Sanger sequencing of sgRNA1–12 sites (*AR*: sgRNA1–8; *DMRT1*: sgRNA9–12). Red boxes mark C→T mutations; matrices show frequencies. (**B**) Agarose gel electrophoresis of *AR* (571 bp) and *DMRT1* (805 bp) PCR products. WT and H_2_O as controls; red arrows indicate expected sizes. (**C**) Editing efficiency statistics. sgRNA6 showed the highest *AR* efficiency (vs. control, *p* < 0.05); sgRNA9 was most efficient for *DMRT1*. Different letters indicate significant differences among groups (*p* < 0.05); *** indicates *p* < 0.001, and ns indicates no significant difference.

After cell transfection and FACS, genomic DNA was extracted from the cell samples obtained from three independent cell transfection experiments. PCR amplification was performed using specific primers at both ends of the editing targets (Figure 3B), and sanger sequencing and editing efficiency analysis were performed on the amplification products (Figure 3A,C). The CBE editing efficiency of 12 designed sgRNAs targeting *AR* and *DMRT1* genes in DF-1 cells was determined with three independent biological replicates (*n* = 3), and the experimental results were statistically analyzed and expressed as the mean ± standard deviation (SD). Different letters (A–D) indicate significant differences among groups (*p* < 0.05) analysis of sequencing chromatograms was further performed to quantify the efficiency of C-to-T base conversion, and the detailed CBE editing efficiency of each sgRNA was confirmed as follows: the editing efficiency of the negative control (NC) 0.00 ± 0.00%, sgRNA1 95.67 ± 2.52%, sgRNA2 91.00 ± 2.00%, sgRNA3 46.00 ± 1.73%, sgRNA4 86.33 ± 9.29%, sgRNA5 42.67 ± 5.51%, sgRNA6 94.67 ± 6.66%, sgRNA7 59.00 ± 6.24%, sgRNA8 37.00 ± 5.29%, sgRNA9 6.67 ± 6.51%, sgRNA10 0.00 ± 0.00%, sgRNA11 0.33 ± 0.58%, and sgRNA12 0.00 ± 0.00%. The sequencing results further revealed that among all the above sgRNAs, the highest C-to-T base conversion efficiencies were achieved with sgRNA1 (95.67 ± 2.52%) and sgRNA9 (6.67 ± 6.51%) in DF-1 cells, respectively, which directly indicated that the CBE plasmids constructed in this study exerted effective editing activity and that the editing efficiency varied significantly due to differences in the edited target loci.

Comparative statistical analysis of the editing efficiency among different sgRNAs was subsequently conducted, and the results showed significant differences in the CBE editing activity of the 12 sgRNAs in DF-1 cells (*p* < 0.05). Specifically, sgRNA1, sgRNA2 and sgRNA6 exhibited extremely significantly superior C-to-T CBE base editing efficiencies relative to all other candidate sgRNAs, and were clustered into the identical top-efficiency statistical group (*p* < 0.001). No statistically significant difference was detected in average editing performance between sgRNA1 and sgRNA6. Among them, sgRNA1 carried the smallest standard deviation (2.52%), which manifested stronger stability and better reproducibility across three independent technical replicates. By contrast, sgRNA6 achieved the peak single-replicate editing efficiency of 99% among all tested sgRNAs, indicating a higher potential upper threshold of its editing catalytic activity. In contrast, sgRNA9, sgRNA10, sgRNA11 and sgRNA12 presented extremely low or no detectable CBE editing efficiency, which was not significantly different from the negative control NC (*p* > 0.05). Based on the above C-to-T base conversion efficiency results and the editing activity characteristics of each sgRNA at specific target loci, sgRNA6 and sgRNA9 were selected as the vectors for subsequent transfection experiments in DF-1 cells and PGCs.

### 3.4. Off-Target Analysis Results

**Figure 4:**
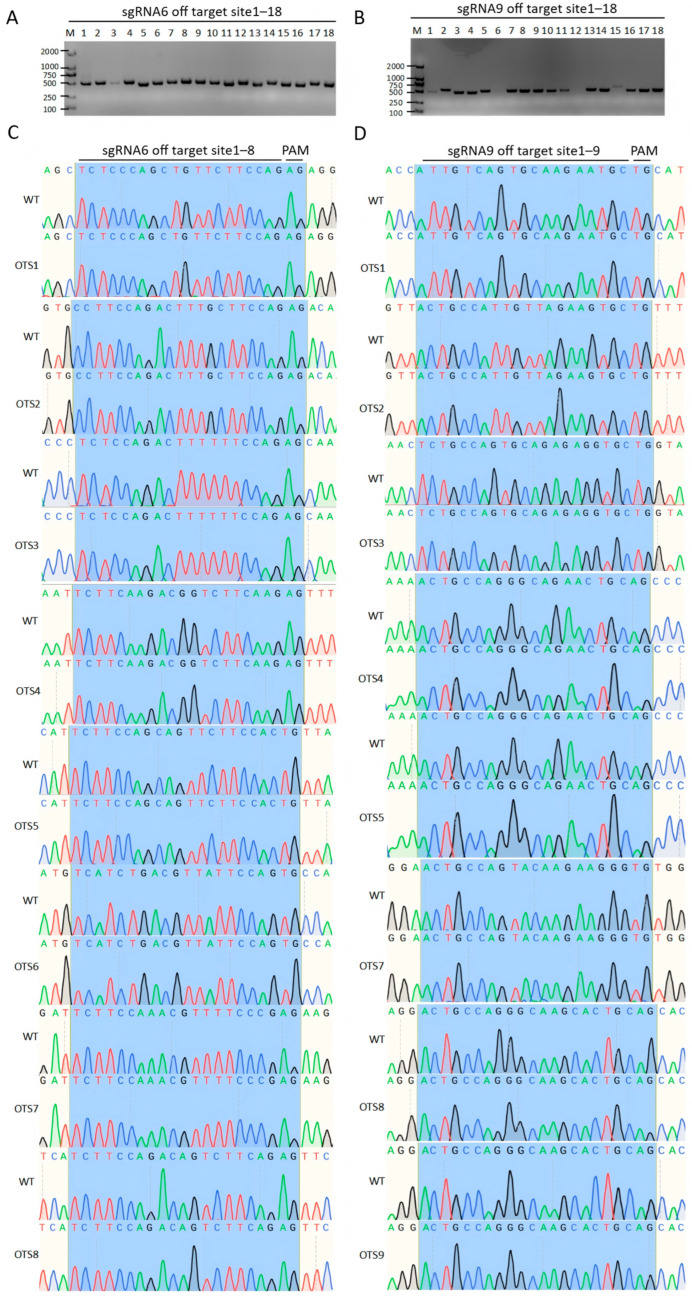
Evaluation of off-target effects of CBE-mediated base editing. (**A**,**B**) Agarose gel electrophoresis of PCR amplicons targeting predicted off-target sites for sgRNA6 (**A**) and sgRNA9 (**B**). All products show a single clear band of 500–600 bp, confirming the specificity of amplification (with OT6 and OT9 for sgRNA9 failing to amplify). (**C**,**D**) sanger sequencing chromatograms of representative high-risk off-target sites for sgRNA6 (**C**) and sgRNA9 (**D**). The editing groups exhibit identical sequences to the WT control, with no C-to-T conversions or indels detected in the sgRNA binding region or PAM.

To validate the editing specificity of sgRNA6 targeting *AR* and sgRNA9 targeting *DMRT1*, we systematically verified 18 predicted high-risk off-target sites for each sgRNA. Genomic fragments corresponding to 36 predicted off-target sites were amplified by PCR, and agarose gel electrophoresis revealed that all products appeared as a single clear band of 500–600 bp (with the exception of OT6 and OT9 for sgRNA9, for which no target amplicons were obtained), with no non-specific bands or amplification artifacts observed, confirming the validity and specificity of the amplification products (Figure 4A,B). Based on homology to the target sequence and genomic conservation, 16 high-risk sites (8 for sgRNA6: OT1–8; 8 for sgRNA9: OT1–8) were selected for sanger sequencing validation (Figure 4C,D). The results showed that the sequencing chromatograms of the cytosine CBE editing groups were completely consistent with those of the wild-type (WT) control group. No unintended modifications, such as C-to-T conversions or insertions and deletions (indels), were detected in the predicted editing regions, including the sgRNA binding site and protospacer adjacent motif (PAM). Sequencing results for the remaining 20 sites (see Supplementary Table S2) also confirmed that the sequences of the editing groups perfectly matched those of the WT control group, with no off-target mutations observed.

### 3.5. Editing Efficiency of Optimized sgRNAs in PGCs

**Figure 5:**
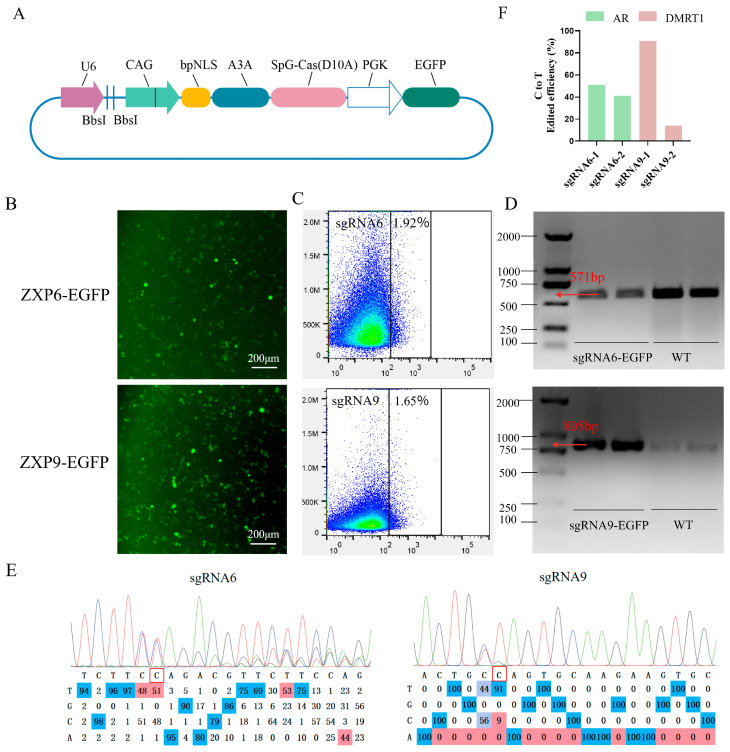
CBE-mediated editing inPGCs. (**A**) Schematic of the CBE-EGFP expression vector. (**B**) *EGFP* fluorescence in PGCs transfected with ZXP6-EGFP and ZXP9-EGFP. (**C**) Flow cytometry analysis of *EGFP*-positive PGCs. (**D**) PCR validation of *AR* and *DMRT1* target regions. (**E**) Sanger sequencing chromatograms of edited loci, the red boxes mark the target cytosine (C) for C→T conversion, which converts the endogenous CAG codon to a premature TAG stop codon. (**F**) Quantified *AR* and *DMRT1* editing efficiency in sorted PGCs.

To validate the efficacy of the CBE system in PGCs, we modified the two most efficient sgRNA plasmids (sgRNA6 and sgRNA9) previously identified in DF-1 cell, generating the recombinant plasmids ZXP6-EGFP and ZXP9-EGFP (Figure 5A). The resulting constructs were then used to transfect PGCs (Figure 5B). Upon fluorescence excitation, prominent green fluorescent signals were detected, confirming the successful transfection of PGCs and the efficient expression of the *EGFP* reporter gene.

FACS was then used to evaluate the transfection efficiency. Our results demonstrated that the maximum transfection efficiencies of sgRNA6 targeting *AR* and sgRNA9 targeting *DMRT1* in PGCs reached 1.92% and 1.65%, respectively (Figure 5C). In the *EGFP* group, a clear population of cells with *EGFP* fluorescence was identified, and all *EGFP*-positive cells were sorted for subsequent experiments. These results demonstrate that the electroporation transfection method followed by flow cytometric sorting can effectively isolate *EGFP*-positive cells from the total cell population, providing a sufficient number of target cells for subsequent studies.

After cell transfection and FACS, genomic DNA was extracted from the cell samples obtained from two independent cell transfection experiments and subjected to PCR amplification (Figure 5D). This further demonstrates the effectiveness of our flow cytometry-based positive cell sorting.

The PCR amplicons were further subjected to sanger sequencing and editing efficiency analysis (Figure 5E). Analysis of sequencing chromatograms was performed to quantify the efficiency of C-to-T base conversion. The sequencing results showed that sgRNA6 targeting *AR* and sgRNA9 targeting *DMRT1* genes, the highest C-to-T base conversion efficiencies were achieved with 51% and 91% in PGCs, respectively (Figure 5F). This is different from the results on DF-1. The increased heterozygous peaks in this positive cell sample were attributed to the inherent heterogeneity of the edited cell population and normal technical fluctuations of the EditR assay, which was consistent with the methodological characteristics of EditR reported previously [27].

## 4. Discussion

As an efficient and precise gene-editing tool that circumvents the limitations of traditional CRISPR/Cas9 systems, such as unintended indels or chromosomal translocations caused by DSBs, CBE has driven innovative progress in the genetic improvement of agricultural species [28]. Although base editing has been validated in a limited number of avian studies [29,30,31,32], our work represents the first systematic application of the CBE to achieve targeted gene knockout of *AR* and *DMRT1* via the introduction of premature stop codons, with validations performed in both chicken somatic cells (DF-1) and PGCs, with the highest editing efficiencies reaching 98% in DF-1 cells and 91% in PGCs, respectively. By inducing precise C-to-T conversions (generating TAG stop codons) without DSB formation, we not only expanded the application scope of CBE in poultry research but also established a standardized, reproducible workflow that can be extended to other avian species and target genes. Efficient CBE editing relies on high-activity sgRNA design and rigorous screening, and our results revealed substantial heterogeneity in sgRNA performance (e.g., sgRNA6 for *AR* with 94.7% ± 6.7% editing in DF-1 vs. sgRNA8 with <40% efficiency), which aligns with previous findings that sgRNA efficacy is shaped by multiple sequence-specific factors, including GC content, PAM-proximal mismatches, and chromatin accessibility [33]. Editing efficiency in chicken cells is jointly regulated by sgRNA sequence characteristics and transfection efficiency, with methodological fluctuations also affecting quantitative outcomes, highlighting the need for concurrent sgRNA screening and transfection condition optimization [34,35,36].

PGCs are considered the optimal vehicle for the preparation of gene-edited chickens because they can be cultured, modified in vitro and transplanted into recipient embryos to achieve germline transmission [37]. In this study, we observed prominent cell type- and target gene-dependent editing divergence mediated by CBEs between PGCs and DF-1 fibroblasts, aligning with our core findings: the average editing efficiencies of targeted *AR* and *DMRT1* genes showed distinct patterns across the two cell types. While DF-1 cells provided a convenient platform for initial sgRNA screening, we observed poor correlation between sgRNA activity in DF-1 cells and final performance in PGCs. This discrepancy may be partially explained by the genomic characteristics of DF-1 cells: as reported previously, DF-1 cells possess a complex, non-diploid karyotype with chromosomal fusions and copy number variations, which can alter sgRNA accessibility and base editor activity [38]. In contrast, primary PGCs maintain a normal diploid genome and germline-specific chromatin state, which likely contributes to the higher editing efficiency observed for *DMRT1*-sgRNA9 in this cell type. Additionally, our editing efficiency analyses were performed on bulk PGC populations, which prevents definitive assessment of zygosity in edited cells. Future work involving clonal PGC lines will be necessary to characterize homozygous/heterozygous editing ratios and confirm the stability of the introduced edits. Meanwhile, the target loci of the *DMRT1* and *AR* genes display inherent differences in chromatin openness, epigenetic modifications and gene expression abundance between the two cell types, which ultimately results in divergent CBE editing efficiency across distinct gene-cell combinations [39,40]. Interestingly, even when the same sgRNA was applied under strictly identical experimental conditions, the editing efficiency in PGCs still exhibited marked fluctuations. The underlying cause of this phenomenon is entirely distinct from that of the aforementioned inter-gene-cell editing efficiency disparity, with the core driver being the intrinsic heterogeneity of the PGC population. As incompletely differentiated germ cells, PGCs naturally exhibit inherent variations in cell cycle progression, epigenetic modification status and metabolic activity among individual cells within the population, even following standardized in vitro culture [41,42]. These cellular traits directly govern the binding efficiency of sgRNA to target DNA and the catalytic activity of CBE editing enzymes, thus yielding heterogeneous editing efficiency at the single-cell level that manifests as significant fluctuations in population-level sequencing assays; minor technical variations in sequencing and quantification serve merely as secondary superimposed factors.

Single-base precision editing of the chicken genome is a key focus in avian biotechnology and livestock genetic breeding; conventional strategies rely on CRISPR/Cas9-ssODN-mediated homologous directed repair (HDR) to generate targeted mutations, but are limited by low HDR activity, especially in PGCs, leading to poor editing efficiency and long screening cycles [43,44,45]. The Roslin Institute reported low HDR efficiencies for chicken ANP32A (9–14% in DF-1, 4–7% in PGCs) and NHE1 (12–18% in DF-1, 5–9% in PGCs) [46,47]. Similarly, a South Korean group achieved only 9–16% editing efficiency in DF-1 and 4–7% in PGCs for the chicken *DMRT1* gene using the same approach, highlighting the bottlenecks of this traditional method [48]. Moreover, positive clone isolation via drug screening and limiting dilution takes 3–5 months, greatly increasing experimental time and cost. In comparison, our CBE-mediated base editing system, which requires neither DNA double-strand breaks, HDR nor exogenous ssODNs, achieved drastically improved efficiencies for *AR* and *DMRT1*: 94.7% ± 6.7% and 6.67 ± 6.51% in DF-1 cells, and 51.0% and 91.0% in PGCs, respectively. Notably, despite the relatively low transfection efficiency of CBE in PGCs, the high editing efficiency achieved herein indicates that once CBE is successfully delivered into PGCs, it can efficiently mediate genomic modification in these cells. Combined with flow sorting, positive edited clones were obtained within 1 month, reducing the experimental cycle by over 80%.

It is widely acknowledged that CRISPR-Cas systems have the potential to cause off-target mutations, sometimes at unexpected frequencies [49,50]. To evaluate potential off-target effects, we examined sixteen near-mismatch gRNA sites corresponding to each gRNA employed. In this study, off-target validation was only performed in DF-1 cells, and no off-target mutations were detected at the predicted high-risk sites by sanger sequencing. These results are consistent with previous reports demonstrating that CBEs have higher precision and safety than traditional CRISPR/Cas9 systems [51,52].

The CBE-mediated knockout models established in both somatic DF-1 cells and germline-competent PGCs also provide novel experimental tools for exploring the molecular mechanisms of *AR* and *DMRT1* in avian male development, enabling subsequent generation of transgenic chickens for in vivo functional validation.

Although this study achieved efficient and precise editing of *AR* and *DMRT1* in chicken cells, some limitations still exist. First, editing efficiency in PGCs can be further improved by optimizing transfection parameters, delivery systems and enzyme engineering. Second, the present study only verified the editing effects at the cellular level, and the in vivo functions of *AR* and *DMRT1* need to be further verified using gene-edited chickens. In addition, the current CBE system is only capable of C-to-T conversion, and the application range can be expanded by combining ABEs and prime editing in the future. Despite these limitations, this study successfully established an efficient and specific CBE editing platform for chicken cells, filling the gap in the application of base-editing technology in avian reproductive gene editing.

In summary, this study successfully established a highly efficient and safe CBE-mediated gene knockout system in chicken DF-1 cells and PGCs by targeting the sex-determination genes *AR* and *DMRT1*. Our findings confirm that CBE can achieve precise single-base editing without double-strand breaks, which fills the gap of base editing application in avian germ cells and provides a reliable technical platform for the preparation of gene-edited chickens. This study promotes the development of precision breeding in poultry. For future research, we will further optimize the delivery efficiency of CBE in PGCs, verify the off-target effects in PGCs at the whole-genome level, and generate gene-edited chickens through germline transmission to explore the in vivo function of *AR* and *DMRT1* genes.

## Figures and Tables

**Table 1:** sgRNA target sequences for chicken *AR* and *DMRT1* genes.

Gene	Name	Wild Type Sequence	Mutant Sequence	Target Site
*AR*	sgRNA1	5′CCCGCAGAGTCCTCGCCCGC3′	5′CCCGTAGAGTCCTCGCCCGC3′	382–401
	sgRNA2	5′CGTTCCAGGCGCCGCGGGA3′	5′CGTTCTAGGCGCCGCGGGA3′	323–342
	sgRNA3	5′TTCCAGGCGCCGCGGGACG3′	5′TTCTAGGCGCCGCGGGACG3′	325–344
	sgRNA4	5′TTCTTCCAGAGCGTGTGCGA3′	5′TTCTTCTAGAGCGTGTGCGA3′	301–321
	sgRNA5	5′CTTCCAGAGCGTGTGCGAAG3′	5′CTTCTAGAGCGTGTGCGAAG3′	304–323
	sgRNA6	5′TCTTCCAGACGTTCTTCCAG3′	5′TCTTCTAGACGTTCTTCCAG3′	291–310
	sgRNA7	5′TTCCAGACGTTCTTCCAGAG3′	5′TTCTAGACGTTCTTCCAGAG3′	293–312
	sgRNA8	5′AGGTGCAGCTGGGGATCGGG3′	5′AGGTGTAGCTGGGGATCGGG3′	231–250
*DMRT1*	sgRNA9	5′ACTGCCAGTGCAAGAAGTGC3′	5′ACTGCTAGTGCAAGAAGTGC3′	224–244
	sgRNA10	5′GGCCGCGCAGGTGAGACGCG3′	5′GGCCGCGTAGGTGAGACGCG3′	259–278
	sgRNA11	5′GCGGCAGCGGGTGATGGCCG3′	5′GCGGTAGCGGGTGATGGCCG3′	274–293
	sgRNA12	5′CGCGCAGGTGAGACGCGGGG3′	5′CGCGTAGGTGAGACGCGGGG3′	277–296

sgRNA1–8 were designed to target exon 1 of the *AR* gene, and sgRNA9–12 were designed to target exon 1 of the d*DMRT1* gene. The wild-type target sequences and corresponding mutant sequences. Predicted base editor targeting bases (positions 4 to 8) are shown in yellow, the mutant base are shown in red, along with the target site positions (nucleotide coordinates) on the respective gene reference sequences.

## Data Availability

The data presented in this study are available upon request from the corresponding author due to the strict management of various data and technical resources within the research teams.

## Supplementary Materials

The following supporting information can be downloaded at: https://www.mdpi.com/article/10.3390/vetsci13050455/s1, Table S1: Primers used for constructing plasmids; Table S2: Off-target sites (OTS) and corresponding primers used for analysis of cytosine base editing of chicken DF1.
