# Supplementary material for: Efficient and Safe Knockout of AR and DMRT1 Mediated by Cytosine Base Editors in Chicken DF-1 and PGCs
Source: Vet Sci. 2026 May 6;13(5):455. doi: 10.3390/vetsci13050455 (PMC13211467; doi:10.3390/vetsci13050455)
Supplement: Supplementary file 1 [file vetsci-13-00455-s001.zip › vetsci-4246812-supplementary.pdf]

Supplementary Material

Supplementary Table S1. Primers used for constructing plasmids

Gene	Primer sequence (5' to 3')	
AR	ZXP1-F:CACCGCCCGCAGAG TCCTCGCCCGC	ZXP1-R:AAACGCGGGCGAGG ACTCTGCGGGC
	ZXP2-F:CACCGCGTTCCAGG CGCCGCGGGA	ZXP2-R:AAACTCCCGCGGCG CCTGGAACGC
	ZXP3-F:CACCGTTCCAGGCG CCGCGGGACG	ZXP3-R:AAACCGTCCCGCGG CGCCTGGAAC
	ZXP4-F:CACCGTTCTTCCAG AGCGTGTGCGA	ZXP4-R:AAACTCGCACACGC TCTGGAAGAAC
	ZXP5-F:CACCGTCTTCCAGA CGTTCTTCCAG	ZXP5-R:AAACCTGGAAGAAC GTCTGGAAGAC
	ZXP6-F:CACCGCTTCCAGAG CGTGTGCGAAG	ZXP6-R:AAACCTTCGCACAC GCTCTGGAAGC
	ZXP7-F:CACCGTTCCAGACG TTCTTCCAGAG	ZXP7-R:AAACCTCTGGAAGA ACGTCTGGAAC
	ZXP8-F:CACCGAGGTGCAGC TGGGGATCGGG	ZXP8-R:AAACCCCGATCCCC AGCTGCACCTC
	ZXP9-F:CACCGACTGCCAGT GCAAGAAGTGC	ZXP9-R:AAACGCACTTCTTG CACTGGCAGTC
DMRT1	ZXP10-F:CACCGCGGCAGCG GGTGATGGCCG	ZXP10-R:AAACCGGCCATCA CCCGCTGCCGC
	ZXP11-F:CACCGGCCGCGCA GGTGAGACGCG	ZXP11-R:AAACCGCGTCTCA CCTGCGCGGCC
	ZXP12-F:CACCGCGCGCAGG TGAGACGCGGGG	ZXP12-R:AAACCCCCGCGTC TCACCTGCGCGC
**Template**	**Primer sequence (5' to 3')**	

**PGK-EGFP-Donor**	F:AGGCATGCTGGGGAGCGGCCGCGGGTAGGGGAGGCGCTTTTC<br>R:CAGGCGCCCCTGCAGGTCGACAGAATTGATCCCCACGCGCC

Supplementary Table S2. Off-target sites (OTS) and corresponding primers used for analysis of cytosine base editing of chicken DF1

**Target Gene**	**OTS No.**	**Off-target site ***	**Primer sequence（5'→3'）**	**Amplicon (bp)**
AR	OT1	TCTTCCAGACGCTCTCCCAGTG	F:CTGCGTTAATGCGTTGGAGG<br>R:CAGGTGAGTTGCTGGAGGG	522
	OT2	ACTTCCAGACTTCTTCCAGGG	F:CGTTTGCAACAGAGGTGCAA<br>R:TGCGCTCTGTTCAAGGTAGG	544
	OT3	TCTCCCAGCTGTTCTTCCAGAG	F:TGTTCCAGCGCTCTGTATCC<br>R:GTTAGCCCTGCCTTGGAAAG	527
	OT4	CCTTCCAGACTTTGCTTCCAGAG	F:GCTGCAATTGGCTGTCACAA<br>R:CCAAGCCTGGTGTCATCTGT	556
	OT5	TCTCCAGACTTTTTTCCAGAG	F:GTGTGCTCAGTTTGCTGCTC<br>R:AGCTCCAACCTTCTTCCACC	505
	OT6	TCTTCAAGACGGTCTTCAAGAG	F:GGACTGGTTGGCTGGAGATG<br>R:GCACTGTTTTGGCATCAGGT	534
	OT7	TCTTCACAAACCTTTTTCCAGGG	F:GGTATCTCTGCTCCTGGGGA<br>R:AAGATCTGCCAGCTCCCAAC	547

OT8	TCTTCCAGCAGTTCTTCCACTG	F:GTGAAGGTGTCAGGAAGCGA<br>R:AGATTCTGGGGCGTTCCATG	559
OT9	TCTTCCAGATGATCATTCCAGAG	F:GGTCTCACAGCCACAAACCT<br>R:ACTTTTAGCCACAACATGCACA	555
O10	TCaTCtGACGTTaTTCCAGTG	F:CCTCTGAAACACCTGGCTGT<br>R:GCCAGGAGCTGTGAGTTCTT	547
OT11	TCACCAGAGGTTCTTCCAGAG	F:AGCAGAGGGTGGATGTACCT<br>R:ATGAGAGCGGATGCAATTGC	506
OT12	TCTTCCAAACGTTTTCCCGAG	F:TGCAAGAAGATTGTCATACTGGC<br>R:AGCTTCACAGTCATGTCCATGT	540
OT13	TCTTCCAGACAGTCTTCAGAG	F:AACAGGAAATGCGGCAATGT<br>R:AGCTGAATTTTGCTGCACAG	503
OT14	TCTTTCAGCTTTCTTCCAGGG	F:TGACCATGTATTTGGACAAAGCA<br>R:CAGGTAGCAGTCCGAAGTGG	553
OT15	TCTTCCAGATTTCTTCCATAG	F:AGAGACCTGCAATTTCCCAGA<br>R:TTGGTCCCACTGCTGTTACC	517
OT16	TCTTCCAGTTGTTCTCCAGAG	F:GACCTCCTTGAAGACCGTGC<br>R:TCCCCTGCAGCGTATTTGTT	511
OT17	TCTTGCAGACTTCTTTCAGTG	F:TTCCCTCATGTGCTAGCAGA<br>R:TCATATCCACATCAGCTGTTGC	559
OT18	TCTGCCCGACTTCTTCCAGGG	F:TTGCTTCCCATCAGCTCGAG<br>R:TGTGGACAAGGTGGGAAGTG	531

	OT1	ACTGCCATTGTTAGAAGTGCTG	F:GCTTTGTCTTGCTGTTGGGG R:TCATCAGTCTTAGAAGGCTCGT	504
	OT2	TCTGCCAGTGCAGAGAGGTGCTG	F:TGGAGCACACTCTAGAGGCT R:GCTCCAAGACCAAATGACAGG	582
	OT3	ACGCCAGTGCAAGAGGTACTG	F:TCCATGTGAAAAGGACGGGT R:GGGTGGTTCTCACTGTATGCA	508
	OT4	ACTGCCAGGGCAGAACTGCAG	F:CAGCATCTTGAGCAACCTGC R:TGTAAAACGCCTCACTGACT	502
	OT5	ACTGCCAGTACAAGAAGGGTG	F:CAAAGAGTGGACGGAGAGCA R:CTGTGCCTCACCACTCTCTG	533
DMRT1	OT6	ACTTCAAGTGCAAGAGGTGCAG	F:GCAAGATGGCCAAGAAGTGC R:CAGAAACAAGCGACACCCCA	508
	OT7	ACTGCCAGGGCAAGCACTGCAG	F:GAAGGTACCATCAAGGCCCC R:TTGTGACTGCCTGTGTCCTC	530
	OT8	ACTGCCAGGGCAAGCACTGCAG	F:AGGAGTCACAATGATGCGGG R:ACTCCAGGCAGAAAGGTCAG	526
	OT9	ACTGCACTGCCAGAAGTGCAG	F:GCACTATGAGCAACAGCAGC R:TGATGTGGCTCCCTTGTGAG	508
	O10	ACAGCCAGCCAAGAAGTGCCG	F:AGCTCTGAGCACAGGGATCT R:ACAGGAAAGTATGGAGAAATAGCTGT	501
	OT11	ATTGTCAGTGCAAGAATGCTG	F:CACCACATCTCCCACCAGTC R:TCCCTTATTCTAACTATTCCCACTGA	518

OT12	ACTGGCAGTGCAAGCTGTGCTG	F:ACCTACATCTATAACTATTCATGCCT R:GGTGGTCCTGAGTTGCTGTT	507
OT13	ACTTCCAGTGCAAGGAGAGCGG	F:ATTTGGCTCTTCTCACCCCC R:TAAGTTTTGCCCGGGGATGT	533
OT14	ATTACCAGTGCAAAAAGTGCAG	F:TACTGGGGAAGCAAACGGTC R:CCTCTGCTGTGAGCTGCATA	523
OT15	ACTGCTGGTGAAGAAGTGCTG	F:AGCAGCAGCCCCTACAAAA R:AATTTCTTTTAAGCATTGCAAAATCCAG	621
OT16	ACTGACAGTGCAAGAGTGAAG	F:TGATGCAGCGCTCTTTCAGA R:TCAGTTACCATGTGTATCAATGCT	528
OT17	TCTGGCACTGCAAGAAGTGCTG	F:CTCACCCTACCTGCTTCTGC R:CAAGCTCCCCGGTTTTTGTG	528
OT18	GCTGCCAGTGCAGGAAGGTGCTG	F:AGACAAGACGAGCACGGTG R:CTCCACTGAGGGCTGAAAGG	538

* The protospacer adjacent motif (PAM) sequence is marked in red.
